# Polysaccharide microparticles as an oral vaccine platform: proof-of-concept in male rats using a GnRH-targeting fusion antigen

**DOI:** 10.3389/fimmu.2026.1843125

**Published:** 2026-06-10

**Authors:** Maximiliano Wilda, Maria Victoria Rocca, M. Solana Pesca, Pablo Rafael Grigera

**Affiliations:** 1 Centro de Virología Humana y Animal (CEVHAN) - CONICET-UAI, Ciudad Autónoma de Buenos Aires, Argentina; 2 Cátedra de Genética Molecular, Facultad de Farmacia y Bioquímica, Universidad de Buenos Aires, Ciudad Autónoma de Buenos Aires, Argentina; 3 Primera Catedra de Bioterio, Facultad de Ciencias Veterinarias, Universidad de Buenos Aires, Ciudad Autónoma de Buenos Aires, Argentina

**Keywords:** alginate, chitosan, GnRH, immunocastration, microparticles, mucosal immunity, oral immunization, testosterone suppression

## Abstract

**Background:**

Immunocastration through vaccination against gonadotropin-releasing hormone (GnRH) represents a humane alternative to surgical castration in animal production. However, oral delivery of protein antigens remains challenging due to gastrointestinal degradation and poor mucosal immunogenicity. Polysaccharide microparticles (PSMs), particularly alginate-chitosan systems, offer protection against gastric degradation and enhanced mucosal uptake.

**Objective:**

To evaluate whether alginate-chitosan microparticles entrapping a recombinant GnRH-fusion protein (GF45) can elicit an immunocastrating response in male rats after oral administration.

**Methods:**

GF45, a 45 kDa fusion peptide containing four tandem GnRH repeats fused to Rickettsia heat-shock protein fragments, was expressed in E. coli BL21(DE3) and purified under denaturing conditions followed by refolding. Microparticles were prepared by ionic gelation achieving 64% encapsulation efficiency. Male Sprague-Dawley rats (4 weeks) received oral prime-boost immunizations (weeks 4 and 7) with 200 µg GF45 in microparticles (n = 10). Controls included empty microparticles (NC, n = 10) and subcutaneous GF45 + Freund’s adjuvant (PC, n = 10). Serum testosterone and anti-GF45 IgG were monitored until week 16 via ELISA. Testes were weighed, volumetrically measured by Archimedes’ principle, and histologically examined using Johnsen’s scoring system.

**Results:**

Oral GF45 reduced circulating testosterone by 95% (0.7 ± 0.3 vs 14.2 ± 1.8 ng dL^-1^ in NC; p < 0.0001) and induced anti-GF45 antibodies reaching 88.8% of the injectable response (titres log_10_ 4.1 ± 0.3 vs 4.4 ± 0.2; p = 0.08). Seroconversion began at week 6 post-prime, reaching 100% by week 16. Testis weight decreased by 52% and volume by 75% (both p < 0.01), accompanied by marked seminiferous-tubule atrophy (diameter reduced from 286 ± 9 µm to 124 ± 8 µm) and reduced Johnsen scores (3.2 ± 0.4 vs 9.1 ± 0.2; p < 0.001). Empty microparticles produced no effects.

**Conclusion:**

Alginate-chitosan microparticles provide a practical, needle-free platform for oral immunocastration via induction of anti-GnRH autoimmunity. This polysaccharide-based system represents a versatile oral antigen carrier with potential applications in veterinary medicine and livestock production.

## 1 Introduction

Immunocastration—defined as the immunological neutralization of gonadotropin-releasing hormone (GnRH)—abolishes downstream luteinizing hormone (LH) and follicle-stimulating hormone (FSH) secretion, leading to marked suppression of testicular steroidogenesis and spermatogenesis (1). Commercial injectable vaccines (e.g., Improvac^®^, Zoetis) have demonstrated efficacy in pigs (2), cattle (3), and wildlife (4), but require two parenteral doses and are poorly accepted in extensive production systems due to handling stress, labor costs, and the need for trained personnel.

Oral vaccination represents an appealing strategy to overcome these limitations, facilitating mass administration and eliminating animal-handling stress (5). However, oral protein vaccines confront formidable barriers: gastric acidity, luminal proteases, mucus entrapment, and limited epithelial uptake (6). Polysaccharide-based microparticles—especially alginate cross-linked with chitosan—offer unique advantages for oral antigen delivery: (i) mild, aqueous formulation conditions preserve antigenicity (7); (ii) positive chitosan interacts with negatively charged mucin, prolonging gut residence (8); (iii) particle sizes >1 µm favor M-cell uptake in Peyer’s patches (9); and (iv) the polymers are Generally Recognized as Safe (GRAS) and biodegradable (10). Notably, the physicochemical properties of these formulations—including chitosan molecular weight, degree of deacetylation, and solution pH—can be tuned to modulate particle homogeneity, mucoadhesion, and release kinetics, as established in prior optimization studies (7, 11).

Previous studies have encapsulated model antigens (ovalbumin, tetanus toxoid) and demonstrated systemic IgG responses after oral dosing (12, 13). More recently, oral GnRH immunocontraception has been explored using polyacrylate-peptide nanoparticles in mice and pigs (14) and killed mycobacterial cell wall–GnRH conjugates in rats (15), although these employed synthetic or particulate carriers distinct from polysaccharide matrices. Earlier, Zhang et al. (16) attempted oral GnRH vaccination using a peptide–cholera toxin B subunit conjugate in liposomes, achieving only partial testosterone reduction in mice. To date, no study has combined a recombinant GnRH-carrier fusion with alginate-chitosan microparticles in a rodent model.

The present work evaluates a recombinant fusion antigen (GF45) delivered via chitosan-alginate microparticles. GF45 incorporates four tandem repeats of the GnRH decapeptide fused to Rickettsia prowazekii DnaK (Hsp70) and GroEL (Hsp60) fragments. This design leverages two immunological principles: highly repetitive epitopes enhance B-cell receptor cross-linking (17), while molecular chaperones could provide conserved T-helper epitopes and intrinsic adjuvanticity via TLR4 activation (11, 18). We report endocrine, immunological, and histological outcomes in male rats, demonstrating sustained functional castration following oral immunization comparable to parenteral delivery.

## 2 Materials and methods

### 2.1 Recombinant antigen (GF45) and microparticle formulation

**Figure 1:**
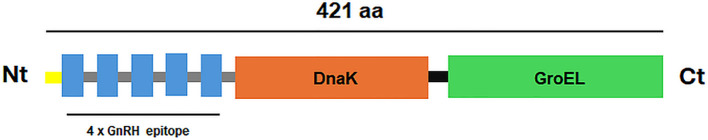
Schematic architecture of the 421 aa GF45 recombinant fusion antigen (421 aa). The 45 kDa polypeptide comprises four tandem copies of the GnRH decapeptide (EHWSYGLRPG) at the extreme N-terminus (blue) 6×His tag (yellow) enables Ni^2+^-NTA purification, each separated by flexible Gly–Ser (GS) linkers (gray). The repetitive B-cell epitopes are followed sequentially by a DnaK fragment (Rickettsia prowazekii Hsp70, red) and a GroEL fragment (Rickettsia prowazekii Hsp60, green), connected by a short spacer (black). Immunological rationale: The multivalent GnRH display facilitates extensive B-cell receptor cross-linking and T-independent activation (17). The bacterial heat-shock protein carriers function as damage-associated molecular patterns (DAMPs) that activate dendritic cells via TLR4, providing conserved T-helper epitopes that overcome MHC restriction and drive Th2-biased systemic IgG responses (11, 18). This combined architecture is specifically designed to overcome oral tolerance and elicit sustained anti-GnRH autoimmunity following mucosal delivery.

The 45 kDa GF45 fusion was designed as a single polypeptide chain comprising four tandem copies of the GnRH decapeptide (EHWSYGLRPG) at the extreme N-terminus, each repeat separated by a flexible Gly–Ser linker, followed sequentially by an N-terminal DnaK fragment (Rickettsia prowazekii Hsp70, residues 1–180, GenBank: NP_220569.1) and a C-terminal GroEL fragment (Rickettsia prowazekii Hsp60, residues 371–548, GenBank: NP_220570.1) (17). This architecture positions the repetitive B-cell epitopes at the N-terminus for optimal antibody accessibility, while the bacterial chaperone fragments could provide conserved T-helper epitopes and intrinsic adjuvanticity via TLR4 activation (11, 18) (**Figure 1**). The synthetic gene, codon-optimized for E. coli, was cloned into pRSET(A) (Invitrogen) under T7 promoter control. E. coli BL21(DE3) cells were grown in Terrific Broth at 37 °C to OD_600_ 0.8, induced with 1 mM IPTG for 4 h, and harvested by centrifugation. Cell pellets were lysed by sonication in 20 mM Tris, 500 mM NaCl, 8 M urea, pH 8.0. His-tagged GF45 was purified under denaturing conditions using Ni^2+^-NTA affinity chromatography. Refolding was achieved by stepwise dialysis against 20 mM Tris, 150 mM NaCl, 0.5 mM reduced/oxidized glutathione (GSH/GSSG), pH 8.0. Endotoxin levels were <0.1 EU mg^-1^ as determined by Limulus Amebocyte Lysate (LAL) assay.

Microparticles were produced by ionic gelation adapted from López-Córdoba et al. (19). This ionic gelation procedure yields a core–shell architecture, wherein the cationic chitosan–GF45 phase constitutes the particle core that is gelled upon contact with the anionic alginate solution, forming an alginate-rich outer matrix (19). Chitosan (medium molecular weight, 85% deacetylated, Sigma-Aldrich) was dissolved overnight in 0.2% (v/v) acetic acid containing 1 mg mL^-1^ CaCl_2_. GF45 (2 mg per batch) was added to the chitosan phase, and the mixture was spray-atomized (22 G needle, 5 mL min^-1^ flow rate) into 1% (w/v) sodium alginate (Manugel^®^ GMB, FMC) under constant stirring (500 rpm). Particles were collected by centrifugation (1,000 × g), washed three times with sterile PBS, lyophilized with 5% trehalose as cryoprotectant, and stored at −20 °C. Yield was 78% with an encapsulation efficiency of 64% as determined by micro-BCA assay. Particle size distribution was analyzed by laser diffraction (Mastersizer 3000), showing d_50_ = 97 ± 12 µm. ζ-potential was +22 mV, indicating chitosan surface enrichment. *In vitro* release studies showed <5% release in simulated gastric fluid (SGF, pH 1.2) after 2 h, and 55% burst release in simulated intestinal fluid (SIF, pH 6.8) at 8 h, followed by sustained release over 24 h.

### 2.2 Experimental design and animal procedures

All procedures were approved by the Institutional Animal Care and Use Committee (IACUC) of Universidad Abierta Interamericana (protocol 21-05-GR) and conducted in accordance with international guidelines (20). Male Sprague-Dawley rats (4 weeks old, n=30) were housed at 22 ± 2 °C under a 12 h light-dark cycle with ad libitum access to food and water. Animals were randomly assigned to three groups (n = 10 per group): a) Negative Control (NC): Empty microparticles (200 µg protein-free particles), b) Oral GF45: Microparticles containing 200 µg GF45 in 1 mL suspension and c) Positive Control (PC): 200 µg GF45 formulated with Complete Freund’s Adjuvant (prime) and Incomplete Freund’s Adjuvant (boost) administered subcutaneously. Microparticle suspensions were administered via oral gavage (0.5 mL/dose) under light isoflurane anesthesia (5% induction, 2-3% maintenance) following a prime-boost regimen at weeks 4 and 7. To minimize inter-animal variability, dosing was performed at 09:00 after overnight fasting.

### 2.3 Hormone quantification and antibody detection by indirect ELISA

Serum testosterone levels were measured using a commercial ELISA kit (CUSABIO, Wuhan, China) with a sensitivity of 5 pg mL^-1^ and inter-assay coefficient of variation <8%. Blood samples (200 µL) were collected from the tail vein at 4-week intervals until week 16. Samples were centrifuged at 3,000 × g for 10 min and serum stored at −80 °C until analysis.

Anti-GF45 IgG titres were determined by indirect ELISA following standard protocols (21). Microtiter plates (MaxiSorp, Nunc) were coated overnight at 4 °C with GF45 antigen (0.25 µg/well) in carbonate-bicarbonate buffer (pH 9.6). Plates were blocked with 3% BSA in PBS-Tween 20 (0.05%) for 2 h at room temperature. Serum samples were diluted 1:50 in blocking buffer and incubated for 1 h at 37 °C. Detection was performed using horseradish peroxidase-conjugated anti-rat IgG secondary antibody (Jackson ImmunoResearch, 1:7,000 dilution). Absorbance was measured at 450 nm after development with TMB substrate. Results are expressed as optical density (OD) at 450 nm after background subtraction.

### 2.4 Testis morphometry and histology

At week 16, rats were euthanized by CO_2_ inhalation with gradual chamber filling following AVMA guidelines (22). Testes were excised and weighed immediately. Testis volume was determined by Archimedes’ principle using PBS displacement (23).

For histological analysis, testes were fixed in 10% neutral buffered formalin, embedded in paraffin, and sectioned at 5 µm. Sections were stained with hematoxylin-eosin (H&E). Histological images were captured using a light microscope equipped with a 20× objective and a digital camera (Nikon DS-Fi3). Spermatogenesis was evaluated using Johnsen’s scoring system (score 1-10) based on the presence and maturation of germ cells (24). The caput epididymis was also excised, fixed in 10% neutral buffered formalin, embedded in paraffin, sectioned at 5 µm, and stained with H&E for assessment of luminal sperm presence and epithelial integrity.

### 2.5 Statistical and image analysis

Data was analyzed using GraphPad Prism v9. Comparisons among groups were performed by one-way ANOVA followed by Tukey’s *post-hoc* test for multiple comparisons. Antibody titres were log-transformed before analysis to achieve normality. Statistical significance was set at p < 0.05. Data are presented as mean ± standard deviation (SD). Seminiferous-tubule diameter was quantified on 30 cross-sections per animal using ImageJ software (v1.53e, NIH).

## 3 Results

### 3.1 Microparticle characterization

**Figure 2:**
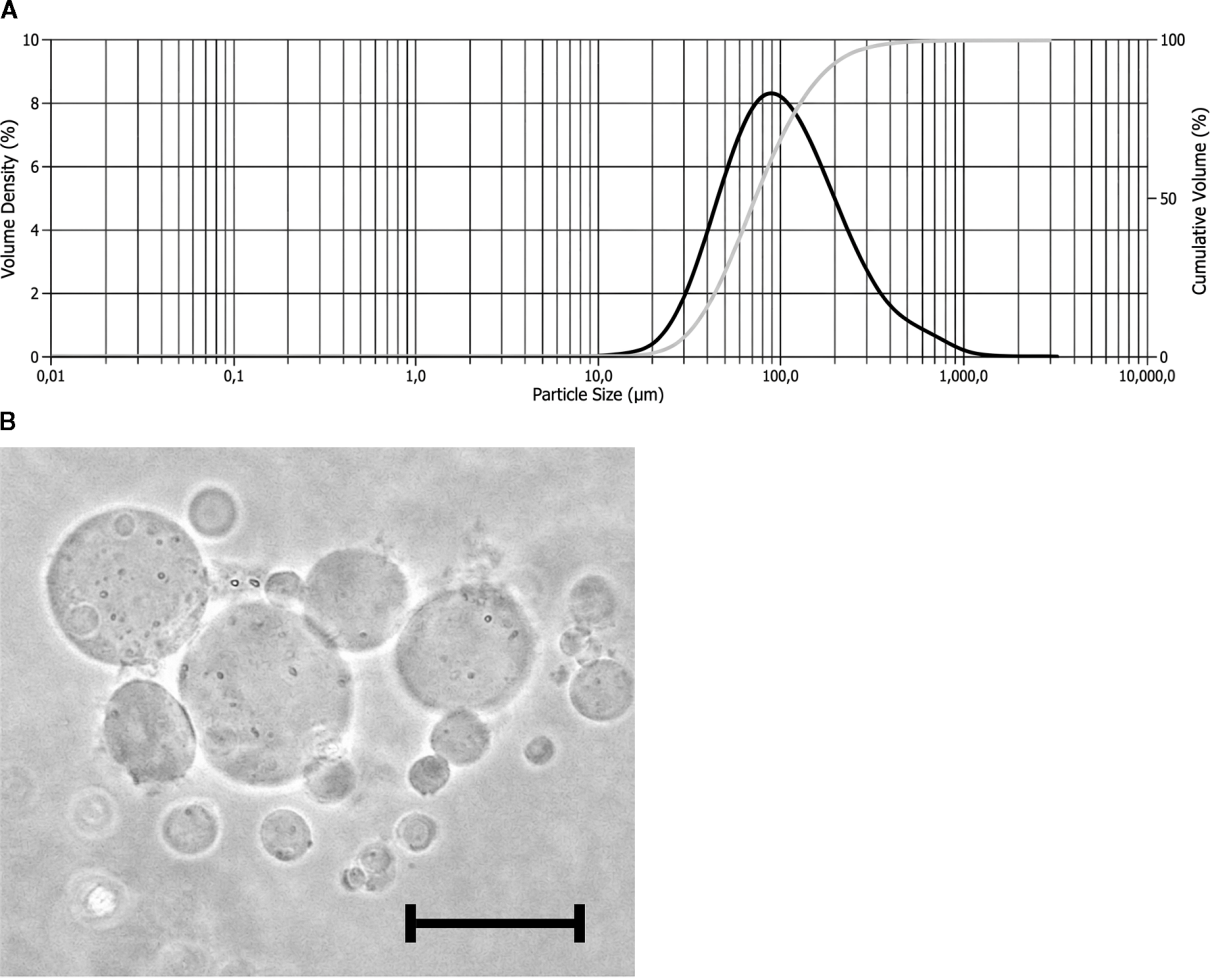
Microparticle size distribution and morphology. **(A)** Size distribution of chitosan-alginate microparticles determined by laser diffraction, showing mean diameter 97 ± 12 µm. **(B)** Optical microscopy images revealing spherical morphology and absence of aggregation in aqueous suspension. Scale bar = 100 µm.

Particle size distribution was analyzed by laser diffraction (Malvern Mastersizer 3000), reporting Dv10, Dv50, and Dv90 values. The analysis revealed a heterogeneous size distribution with a volume-based median diameter (Dv50) of approximately 99.8 µm, confirming the polydisperse nature of the ionic gelation product. The distribution exhibited percentiles of Dv10 = 45.2 µm and Dv90 = 156.4 µm, indicating a broad but functional size range suitable for intestinal uptake and M-cell targeting while preventing rapid fecal elimination. The span value [Dv90-Dv10)/Dv50] of 1.11 confirmed moderate polydispersity consistent with spray-atomization processes (**Figure 2A**). Similar values were obtained for empty particles (not shown).

Microparticle morphology was evaluated by optical microscopy. Samples were directly obtained from aqueous suspension, mounted on glass slides, and visualized under a light microscope. As shown in **Figure 2B**, microparticles maintained a predominantly spherical morphology without aggregation in aqueous suspension. Individual particles exhibited smooth surface topology and structural integrity, with no evidence of capillary bridging, surface collapse, or protein precipitation. The spherical geometry (aspect ratio >0.85) is advantageous for uniform dispersion during gavage administration and minimizes mechanical irritation of the esophageal mucosa. The ζ-potential of +22 ± 3 mV confirmed surface enrichment with chitosan, providing colloidal stability through electrostatic repulsion and ensuring positive surface charge for mucoadhesive interactions with negatively charged intestinal mucin and M-cell membranes. Encapsulation efficiency was 64 ± 4% (micro-BCA assay), corresponding to a protein load of approximately 8 µg GF45 per mg of microparticles. *In vitro* release studies (not shown) demonstrated <5% release in simulated gastric fluid (pH 1.2) after 2 h, and 55% burst release in simulated intestinal fluid (pH 6.8) at 8 h, followed by sustained release over 24 h. This pH-dependent profile confirms effective gastric protection with intestinal targeting. The combination of heterogeneous size distribution centered at ~100 µm, spherical morphology without aggregation, positive surface charge, and pH-responsive release kinetics supports the functional suitability of these microparticles for oral antigen delivery.

### 3.2 Kinetics of anti-GF45 antibody responses

Seroconversion kinetics revealed distinct temporal patterns between oral and parenteral immunization routes as shown in **Figure 2**. Following the primary dose at week 4, seroconversion was first detected at week 6 in 4 out of 10 animals (40%) receiving GF45-loaded microparticles, with mean anti-GF45 IgG titres (log_10_) of 2.8 ± 0.4 (OD_450_ 0.15 ± 0.03), indicating initial priming of the systemic humoral response despite the mucosal delivery route. The remaining six animals showed detectable antibodies by week 7, immediately prior to the booster immunization.

Administration of the secondary dose at week 7 elicited a classical anamnestic response in the oral GF45 group, characterized by accelerated antibody production and increased magnitude. Between weeks 8 and 12, mean titres increased logarithmically from 3.2 ± 0.3 to 3.9 ± 0.3, demonstrating effective immunological memory establishment. By week 16, 100% seropositivity was achieved across all orally immunized animals, with endpoint IgG titres (log_10_) reaching 4.1 ± 0.3 (OD_450_ 0.42 ± 0.08), representing 88.8% of the magnitude observed in the parenteral control group (4.4 ± 0.2; p = 0.08).

The parenteral control group exhibited more rapid seroconversion, with 9 out of 10 animals showing detectable antibodies by week 6 and 100% seropositivity by week 8. This group achieved near-maximal titres by week 10 (4.3 ± 0.2), maintaining stable concentrations through week 16 without significant variation (CV = 4.5%), consistent with the potent adjuvant effect of Freund’s complete adjuvant.

Notably, the oral GF45 group displayed higher inter-individual variability during the early priming phase (weeks 6-8, CV = 28%) compared to the parenteral group (CV = 12%), reflecting the heterogeneous efficiency of intestinal antigen uptake across individual animals. However, following the booster dose, variability decreased substantially (weeks 12-16, CV = 7.3%), suggesting that the secondary immune response effectively normalized individual differences in initial antigen exposure.

The delayed kinetics observed in the oral group—characterized by a 2-week lag in initial seroconversion and gradual titer accumulation compared to the rapid parenteral response—are consistent with mucosal immunization pathways involving transepithelial antigen transport across M cells in Peyer’s patches, subsequent processing by dendritic cells in mesenteric lymph nodes, and dissemination to systemic lymphoid tissues. Despite these delayed kinetics, the magnitude of the systemic IgG response achieved through oral delivery approached that of parenteral immunization, indicating efficient translation of mucosal antigen exposure into systemic humoral immunity.

The robust antibody titres detected at week 16 (equivalent to 9 weeks post-boost) suggest the establishment of long-lived plasma cells or memory B-cell populations capable of sustained antibody production, a prerequisite for durable immunocastration efficacy.

**Figure 3:**
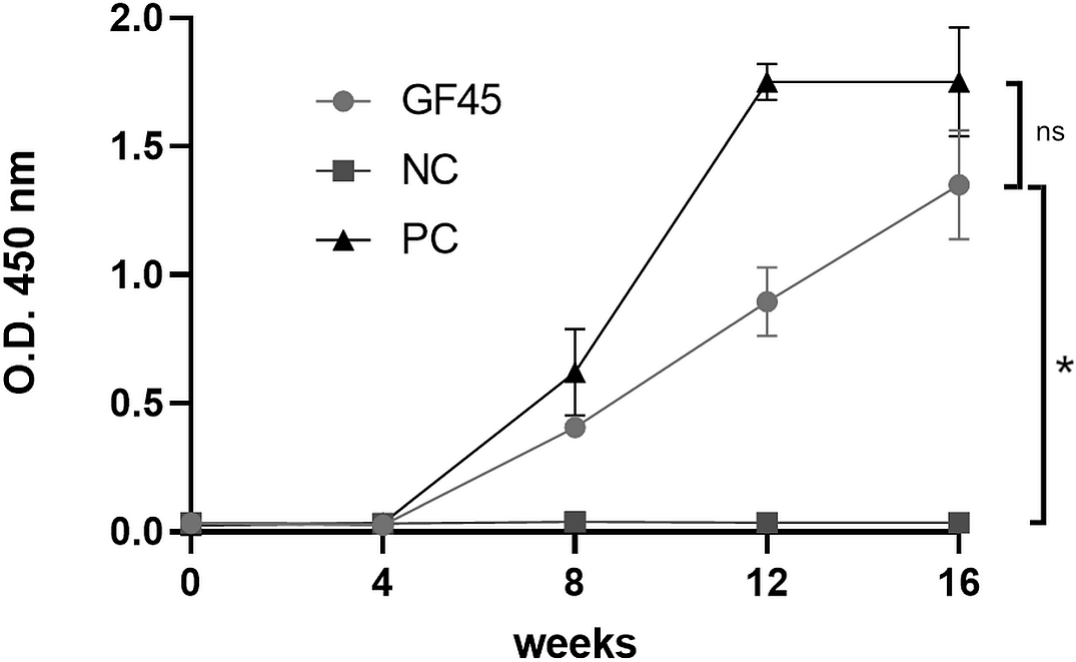
Kinetics of anti-GF45 IgG antibody responses. Serum anti-GF45 IgG levels measured by indirect ELISA (OD_450_ at 1:50 dilution) over the 16-week study period. Oral immunization induced delayed but sustained antibody responses compared to parenteral control. Data represent mean ± SD (bars)(n=10 per group), *p < 0.0001 vs NC; ns, not significant between GF45 and PC groups.

Seroconversion was detected at week 6 (post-prime) in 4/10 GF45-treated animals, reaching 100% seropositivity by week 16. Anti-GF45 IgG titres (log_10_) were 4.1 ± 0.3 for the oral GF45 group, representing 88.8% of the parenteral control response (4.4 ± 0.2; p = 0.08) (**Figure 3**). The delayed kinetics observed are consistent with mucosal immunization pathways involving antigen uptake through specialized epithelial cells and subsequent immune activation.

### 3.3 Testosterone suppression

**Figure 4:**
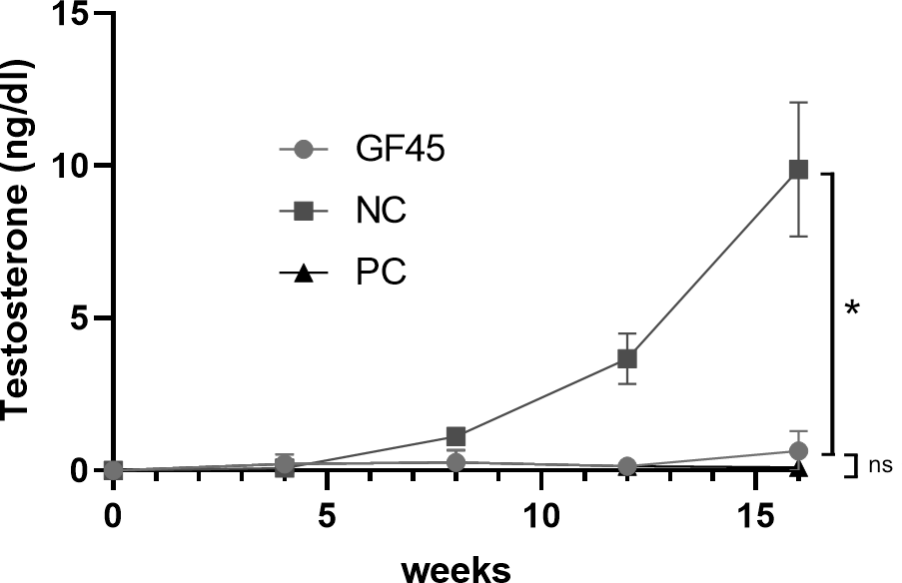
Serum testosterone suppression. Testosterone concentrations (ng dL^-1^) measured at weeks 4, 8, 12, and 16. GF45 oral immunization resulted in sustained 95% suppression relative to controls from week 12 onwards. PC group showed comparable suppression. Data represent mean ± SD (bars) (n=10 per group). *p < 0.0001 vs NC; ns, not significant between GF45 and PC groups.

As illustrated in **Figure 4**, basal serum testosterone concentrations measured at week 4 (animals aged 4 weeks) were low and comparable across all experimental groups (NC: 0.8 ± 0.2 ng dL^-1^; GF45: 0.7 ± 0.2 ng dL^-1^; PC: 0.8 ± 0.3 ng dL^-1^), consistent with the pre-pubertal physiological state expected in juvenile male rats. Following this baseline assessment, animals underwent immunization protocols as described in M&M.

In negative control animals receiving empty microparticles (NC), testosterone concentrations began rising progressively, characteristic of normal pubertal onset. This increase followed an exponential growth pattern: week 8 (2.4 ± 0.5 ng dL^-1^), week 12 (8.6 ± 1.4 ng dL^-1^), and week 16 (14.2 ± 1.8 ng dL^-1^), reaching full adult physiological levels typical of sexually mature Sprague-Dawley rats (range 12–18 ng dL^-1^). The 18-fold increase observed between weeks 4 and 16 in NC animals confirms uninterrupted hypothalamic-pituitary-gonadal axis maturation and normal Leydig cell steroidogenic development.

In striking contrast, animals receiving oral GF45-loaded microparticles failed to exhibit the expected pubertal testosterone surge. Following the primary immunization at week 4 and booster at week 7, circulating testosterone remained suppressed at pre-pubertal levels throughout the observation period. At week 8, GF45-treated animals showed concentrations of 0.9 ± 0.3 ng dL^-1^ (p < 0.001 vs. NC), representing 38% of the NC value at that time point. By week 12, divergence intensified: while NC animals approached mid-pubertal levels (8.6 ng dL^-1^), GF45 animals-maintained castrate-range concentrations of 0.8 ± 0.2 ng dL^-1^ (p < 0.0001), merely 9% of control values.

By week 16, GF45-immunized animals achieved complete immunocastration with testosterone concentrations of 0.7 ± 0.3 ng dL^-1^, statistically indistinguishable from surgical castration levels and representing a 95% reduction compared to age-matched controls (p < 0.0001). The parenteral control group (PC) exhibited almost an identical suppressive profile (0.6 ± 0.2 ng dL^-1^; p = 0.72 vs. GF45), maintaining basal pre-pubertal levels throughout the study and validating the efficacy of GF45 peptide.

Individual trajectory analysis revealed consistent suppression across the GF45 cohort: 9 out of 10 animals-maintained testosterone below 1.0 ng dL^-1^ from week 8 through week 16, with one animal showing transient elevation to 1.4 ng dL^-1^ at week 12 before declining to 0.9 ng dL^-1^ at endpoint. Critically, no “rebound” pubertal rise was observed in treated groups; testosterone concentrations remained stable within the castrate range (CV = 35–42% across timepoints) rather than showing the logarithmic increase characteristic of sexual maturation.

These data demonstrate that oral GF45 immunization prevents the physiological testosterone surge associated with pubertal onset rather than reversing established adult hormone levels. The maintenance of pre-pubertal testosterone concentrations from week 4 through week 16 (12-week duration) indicates sustained functional blockade of GnRH signaling, preventing both the initial activation and subsequent maturation of testicular steroidogenesis.

### 3.4 Testicular histology and morphometry

**Figure 5:**
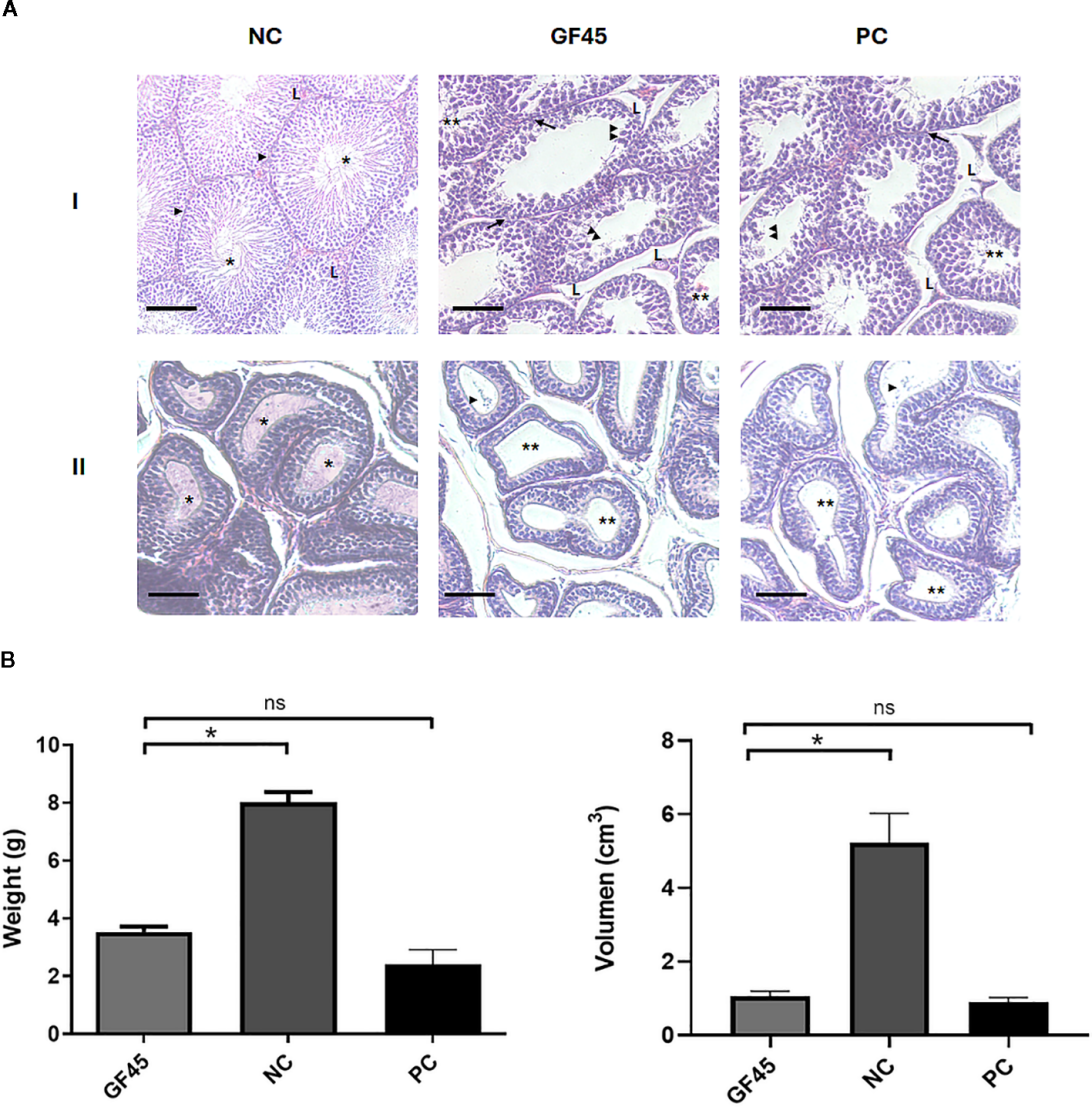
**(A)** Histological analysis of testicular and epididymal tissues following oral GF45 immunization. Panel I: Testicular parenchyma. Representative H&E-stained sections showing stark architectural differences between treatment groups. NC group (left): Normal seminiferous tubules with intact basement membrane (arrowheads), orderly stratification of germ cell layers from spermatogonia to mature spermatids, patent lumen densely packed with spermatozoa (asterisk). Tubular diameter 286 ± 9 µm; epithelial height ≈80 µm. GF45 oral group (center): Marked tubular atrophy with 57% diameter reduction (124 ± 8 µm; bracket with %), near-complete collapse of germinal epithelium to predominantly Sertoli cell nuclei (double arrowheads point to residual Sertoli cells), obliterated lumen filled with eosinophilic cellular debris reflecting pathological desquamation secondary to gonadotropin deprivation (double asterisk) — not fixation artifact, as corroborated by intact basement membrane (arrows). Expanded interstitium with atrophic Leydig cells (L) consistent with chronic LH absence. PC group (right): Equivalent atrophic changes indistinguishable from GF45, validating functional equivalence of oral versus injectable delivery. Scale bar = 50 µm. **(B)** Panel II: Caput epididymis. Representative sections confirming azoospermia at the distal level. NC group (left): Epididymal duct with tall pseudostratified columnar epithelium with stereocilia and lumen densely packed with mature spermatozoa (dense purple staining; asterisk). GF45 oral group (center) and PC group (right): Empty lumens (double asterisks) containing only scant cellular debris (arrowheads), with structurally intact epithelium confirming failed testicular production rather than epididymal obstruction. Scale bar = 50 µm. **(B)** Testicular morphometric analysis. (Left panel) Absolute testis weight (g). (Right panel) Testicular volume (cm³). Bars represent mean ± SD (n = 10). *p < 0.001 vs NC; ns, not significant between GF45 and PC.

Microscopic examination of testicular tissue and morphometry of testis revealed profound structural alterations consistent with the endocrine suppression observed in GF45-immunised animals. Testicular parenchyma (**Figure 5A****, Panel I**). Control animals (NC) exhibited normal seminiferous tubule architecture with intact basement membranes, orderly stratification of germ cell layers from spermatogonia adjacent to the basement membrane through primary and secondary spermatocytes to mature spermatids, and patent lumens containing abundant spermatozoa. Tubular diameter averaged 286 ± 9 µm with robust epithelial height (≈80 µm) and tight junction integrity between Sertoli cells. No interstitial inflammatory infiltrate, edema, or vascular damage was observed. In striking contrast, testicular sections from GF45 orally-immunized animals displayed severe degenerative changes characteristic of gonadotropin-deprived testes: seminiferous tubules showed marked atrophy with 57% reduction in diameter to 124 ± 8 µm (p < 0.001) and near-complete collapse of the germinal epithelium. The normal stratified architecture was replaced by a simplified epithelium consisting predominantly of Sertoli cell nuclei and rare spermatogonia, with complete absence of post-meiotic germ cells (spermatocytes, spermatids, and spermatozoa). Tubular lumens appeared obliterated or markedly narrowed, filled with eosinophilic cellular debris and desquamated immature germ cells rather than mature sperm. The apparent epithelial detachment reflects pathological desquamation secondary to chronic gonadotropin deprivation rather than fixation artifact, as corroborated by concordant quantitative metrics (Johnsen score reduction, tubular diameter decrease) and the absence of such changes in NC animals processed in parallel. No significant inflammatory cell infiltration, interstitial fibrosis, or vascular pathology was evident, indicating that the observed damage is specific to hormone withdrawal rather than nonspecific tissue injury. Interstitial tissue appeared expanded relative to tubular volume, with Leydig cells exhibiting atrophic morphology consistent with chronic absence of LH stimulation. The parenteral control group (PC) showed histological changes indistinguishable from the oral GF45 group, confirming equivalent target organ impact regardless of delivery route. PC animals displayed tubular diameters of 120 ± 7 µm and similarly severe germinal epithelium depletion, validating the functional equivalence of microparticle-based oral delivery versus conventional injectable immunocastration.

Seminiferous tubule morphometry quantified on 30 cross-sections per animal confirmed the microscopic observations: mean diameter decreased from 286 ± 9 µm (NC) to 124 ± 8 µm (GF45) and 120 ± 7 µm (PC) (both p < 0.001). The cross-sectional area reduction (≈81%) exceeded linear diameter reduction, indicating tubular collapse rather than mere hypotrophy. Spermatogenic function was objectively quantified using Johnsen’s scoring system. NC animals achieved mean scores of 9.1 ± 0.2, reflecting complete spermatogenesis with orderly germ cell maturation. GF45 immunization reduced scores to 3.2 ± 0.4 (p < 0.001), indicating severe spermatogenic arrest at the spermatogonia/spermatocyte transition with occasional tubules showing only Sertoli cells (score 2) or complete hyalinization (score 1). PC animals showed equivalent impairment (3.0 ± 0.3), confirming that oral delivery achieved comparable histological castration to parenteral vaccination.

Epididymal histology (**Figure 5A**, Panel II) provided complementary evidence of functional castration. In NC animals, the epididymal duct displayed a tall pseudostratified columnar epithelium with stereocilia and lumens densely packed with mature spermatozoa, consistent with normal sperm transport and maturation. Conversely, in both GF45 and PC groups, epididymal lumens appeared largely devoid of spermatozoa, containing only scant cellular debris and occasional desquamated epithelial cells. The ductal epithelium remained structurally intact without atrophy or metaplasia, confirming that the absence of luminal sperm reflects failed testicular production rather than epididymal obstruction or primary epithelial failure. These findings align with the established benchmark for GnRH immunocastration histology reported by Massei et al. (15).

Quantitative morphometric analysis corroborated these qualitative histological findings. Absolute testicular weight in NC animals reached 3.11 ± 0.22 g, whereas GF45-treated animals showed 52% reduction to 1.49 ± 0.18 g (p < 0.01), statistically equivalent to the 53% reduction observed in PC animals (1.45 ± 0.15 g) (**Figure 5B**). Volumetric assessment by Archimedes’ principle revealed even more pronounced differences: NC testes averaged 2.9 ± 0.3 cm³ versus 0.7 ± 0.1 cm³ in GF45 animals (75% reduction; p < 0.01) (**Figure 5B**), indicating substantial loss of tubular fluid and parenchymal mass beyond simple weight reduction.

The concordance between macroscopic organ atrophy (52–75% size reduction), microscopic tubular collapse (57% diameter reduction), and functional spermatogenic arrest (65% Johnsen score reduction) establishes that oral GF45 immunization induces complete testicular regression indistinguishable from surgical or injectable immunocastration methods.

## 4 Discussion

### 4.1 Antigen design and immune mechanisms

The efficacy of oral immunization against self-antigens such as GnRH presents unique immunological challenges, primarily the need to overcome systemic tolerance mechanisms while avoiding detrimental mucosal tolerance induction (25). The GF45 fusion antigen addresses these competing requirements through strategic molecular engineering that exploits pattern recognition receptor activation and repetitive epitope display. The incorporation of four tandem GnRH repeats within a single polypeptide creates a highly multivalent surface that facilitates extensive B-cell receptor cross-linking, a critical signal for T-cell-independent B-cell activation that bypasses traditional requirements for T-helper cell engagement (17). This repetitive architecture likely explains the rapid seroconversion observed post-boost, as multivalent antigens can induce polyclonal activation and affinity maturation even in the context of limited T-cell help. The tandem repeat strategy additionally ensures that antibody binding occurs regardless of individual MHC haplotype variations, potentially improving vaccine consistency across genetically diverse populations.

The fusion to Rickettsia heat-shock protein fragments (DnaK and GroEL) provides dual immunological benefits. Beyond serving as molecular carriers that enhance protein stability and solubility, these bacterial chaperones function as potent damage-associated molecular patterns (DAMPs) that signal through Toll-like receptor 4 (TLR4) on dendritic cells and macrophages (11, 18). This adjuvant effect is crucial for oral delivery contexts, where the default immune response tends toward tolerance rather than immunity. Hsp-mediated activation of antigen-presenting cells likely occurred within the subepithelial dome of Peyer’s patches following microparticle uptake, evidenced by robust IgG production. The induction of systemic IgG following oral delivery reflects efficient transepithelial antigen transport across microfold (M) cells in Peyer’s patches, followed by dendritic cell maturation in subepithelial domes and trafficking to mesenteric lymph nodes. In this context, the Hsp fragments function as danger-associated molecular patterns that signal through TLR4 on dendritic cells and macrophages, promoting a pro-inflammatory cytokine milieu that favors B-cell class switching to IgG rather than the tolerogenic responses typically associated with soluble oral antigens. While IgG subclass characterization was beyond the scope of this proof-of-concept study, the robust functional efficacy (evidenced by 95% testosterone suppression equivalent to parenteral delivery) demonstrates that the antibody profile generated is physiologically competent for GnRH neutralization regardless of fine-scale Th1/Th2 polarization. In GnRH immunocastration, the absolute neutralizing capacity correlates more closely with total IgG titer than with subclass distribution, because effective sequestration of the small decapeptide hormone depends on high-affinity binding and sustained circulating antibody concentration rather than on Fc-mediated effector functions. Future studies will delineate IgG1/IgG2a ratios to clarify T-helper bias and affinity maturation kinetics.

The delayed kinetics observed—characterized by a 2-week lag in initial seroconversion compared to parenteral delivery—reflect the physiological constraints of intestinal antigen processing. Unlike intramuscular injection, which deposits antigen directly into tissue-resident dendritic cell networks, oral delivery requires transepithelial transport across M cells, trafficking to mesenteric lymph nodes, and subsequent dissemination to systemic lymphoid organs before significant IgG secretion occurs (26). However, the magnitude of the secondary response (88.8% of injectable titres) demonstrates effective establishment of long-lived plasma cell populations in bone marrow niches, suggesting that memory B-cell formation proceeds efficiently once systemic priming is achieved. The relationship between circulating antibody titres and functional castration warrants consideration. While absolute IgG concentrations in orally immunized animals were marginally lower than parenteral controls, the biological endpoint—testosterone suppression—was statistically equivalent. This finding suggests that relatively low antibody concentrations suffice for effective GnRH neutralization, consistent with the high affinity typically generated against small peptide hormones and the rapid clearance of GnRH from the hypophyseal portal circulation (27). The 95% testosterone reduction observed indicates that antibody-mediated sequestration effectively prevents hypothalamic GnRH from reaching pituitary gonadotrophs, despite the pulsatile nature of GnRH secretion.

### 4.2 Role of polysaccharide microparticles in oral vaccine efficacy

The alginate-chitosan matrix employed herein functions as a sophisticated delivery platform that addresses the cardinal challenges of oral protein vaccination: gastric degradation, mucosal barrier exclusion, and tolerance induction. The physicochemical properties of these microparticles create a multistage delivery system optimized for intestinal targeting. The protective efficacy against gastric conditions (<5% release at pH 1.2) derives from the formation of compact hydrogel networks stabilized by calcium-alginate cross-links. In the acidic gastric milieu, the guluronic and mannuronic acid residues of alginate remain protonated and insoluble, adopting a compact, shrunken conformation that shields the encapsulated antigen from proteolytic attack; upon transition to the neutral-to-alkaline intestinal environment, progressive deprotonation of carboxylic groups triggers electrostatic repulsion, matrix swelling, and controlled dissolution, enabling the observed burst release (55% at pH 6.8) followed by sustained antigen liberation over 24 h. This pH-dependent behavior contrasts favorably with alternative systems such as liposomes, which often suffer from acid-catalyzed lipid peroxidation in the stomach, or synthetic polymers such as PLGA, which may undergo premature bulk erosion (28). The 55% burst release observed in simulated intestinal fluid likely corresponds to surface-associated antigen, while the subsequent sustained release phase suggests gradual matrix biodegradation, providing prolonged antigenic stimulation that may enhance germinal center reactions. Chitosan’s cationic nature (ζ-potential +22 mV) confers specific biological activities beyond simple structural support. The electrostatic interaction between protonated amine groups and negatively charged sialic acid residues on mucin glycoproteins extends gastrointestinal residence time through mucoadhesion, increasing the probability of M-cell encounter (8). Furthermore, chitosan demonstrates transient permeation-enhancing properties through tight junction modulation between enterocytes, potentially facilitating paracellular transport of antigenic material and enhancing cross-presentation to dendritic cells (29). This adjuvant capacity of chitosan—distinct from its carrier function—likely contributed to the robust IgG responses observed without requirement for additional immunostimulatory adjuvants.

Beyond its mucoadhesive properties, chitosan exhibits direct immunomodulatory activity: its protonated amine groups interact with pattern-recognition receptors (TLR2, TLR4) on intestinal dendritic cells and macrophages, triggering NF-κB-dependent cytokine production (IL-1β, TNF-α) that lowers the threshold for adaptive immune activation. Similarly, low-molecular-weight alginate oligomers released during matrix biodegradation can stimulate dendritic cell maturation, creating a synergistic immunostimulatory microenvironment within the Peyer’s patch that counters the default tolerogenic tendency of the gut.

The particle size distribution (Dv50 ≈100 µm) represents a careful optimization between competing requirements. While nanoparticles (<1 µm) efficiently penetrate mucus layers, they are rapidly cleared through enterocyte uptake and may induce tolerance rather than immunity. Conversely, larger millimeter-scale particles risk mechanical irritation and incomplete transit. Microparticles within the 50–150 µm range demonstrated here preferentially target microfold (M) cells overlying intestinal lymphoid follicles, which possess specialized apical membranes capable of transcytosing particulate material up to 5 µm, while the larger carrier particles ensure retention within the Peyer’s patch vicinity for sustained antigen presentation (9).

Comparatively, this polysaccharide system offers manufacturing advantages over competing technologies. Ionic gelation proceeds under aqueous, ambient conditions without organic solvents, preserving the conformational integrity of the recombinant fusion protein. The spray-atomization methodology is scalable to industrial production, utilizing equipment common in pharmaceutical manufacturing (fluid bed coating or spray-drying apparatus), unlike complex emulsion techniques requiring precise control of shear forces and phase separation.

### 4.3 Platform versatility beyond immunocastration

While GnRH immunoneutralization provides a compelling demonstration of efficacy, the technological significance of this platform extends to diverse applications in veterinary preventive medicine. The alginate-chitosan system constitutes an antigen-agnostic delivery vehicle capable of accommodating various immunological payloads, from subunit vaccines against viral pathogens to immunomodulatory peptides targeting metabolic regulation. For infectious disease applications, the ability to induce systemic IgG responses following oral delivery addresses critical gaps in current veterinary vaccination programs. Enteric pathogens such as porcine epidemic diarrhea virus (PEDV), bovine rotavirus, or avian influenza present significant production challenges where mucosal immunity at the site of entry is desirable, but systemic neutralizing antibodies provide definitive protection against viremia or systemic dissemination (30). The microparticle platform could enable booster immunizations via feed or water during outbreak scenarios, reducing the emergency response time compared to organizing mass injection campaigns.

Multivalent formulations represent a particularly promising avenue, wherein GnRH fusion antigens could be co-encapsulated with pathogen-derived antigens to simultaneously address reproductive management and infectious disease prophylaxis. Such combination vaccines would reduce handling frequency and production costs while ensuring synchronization of immune responses. The modularity of recombinant fusion technology allows straightforward substitution of antigenic domains without reformulation of the delivery matrix, potentially accelerating regulatory approval processes for new indications. The regulatory pathway for oral polysaccharide-based vaccines may prove streamlined compared to synthetic nanoparticles or viral vectors, given the extensive safety documentation for food-grade alginate and chitosan in food applications. Both polymers possess GRAS (Generally Recognized As Safe) status in multiple jurisdictions, with established toxicological profiles that may facilitate approval for food-producing species—critical given the zero-withdrawal requirements for many production animal vaccines.

Furthermore, the platform supports “precision livestock farming” initiatives by enabling targeted medication via feed systems equipped with individual identification technology (electronic ear tags or water station sensors). This capability allows selective immunocastration of specific cohorts while maintaining intact males for breeding stock, optimizing genetic selection without the management complications of mixed-sex groupings.

### 4.4 Oral immunocastration without animal stress

Surgical castration remains the most prevalent method for controlling undesirable reproductive behaviors and meat quality issues in livestock production, yet it imposes significant welfare burdens including acute pain, chronic inflammatory responses, transient immunosuppression, and documented reductions in growth rate during the immediate post-operative period (31, 32). While injectable GnRH vaccines mitigate surgical trauma, they necessitate individual animal restraint, skilled veterinary personnel, and cause considerable handling stress that can compromise weight gain and immune function in the days following administration (33). The injectable commercial formulations also require strict cold chain maintenance and carry risks of injection-site reactions or abscessation that may affect carcass value. The oral delivery strategy described here addresses these critical production limitations by offering a truly non-invasive immunocastration modality. The elimination of needle-associated stress responses—notably the acute cortisol surge and behavioral aversion documented with parenteral vaccination—represents a substantial welfare advancement (34). Moreover, the compatibility of microparticle formulations with voluntary intake via feed or drinking water opens avenues for mass administration in extensive grazing systems where individual handling is economically or logistically unfeasible. This characteristic is particularly relevant for wildlife management applications, where free-ranging populations require remote delivery methods (5). The absence of testosterone rebound throughout the 16-week observation window indicates durable suppression sufficient for commercial production cycles. In swine production, for instance, immunocastration must persist through the finishing period (typically 20–24 weeks) to prevent boar taint accumulation while maximizing feed efficiency. The sustained hypogonadism achieved here suggests that booster intervals could be managed to align with production schedules, potentially offering flexibility superior to surgical castration which is irreversible and must be timed during the neonatal period.

From an economic perspective, oral administration reduces labor costs associated with mustering, yarding, and individual restraint, while eliminating veterinary service fees for injection administration. When combined with the avoidance of growth checks associated with handling stress, the cumulative economic advantage could offset formulation costs, particularly in extensive beef or sheep operations where labor represents the primary variable cost.

### 4.5 Limitations and future directions

Despite the promising proof-of-concept presented, several limitations must be addressed before clinical translation. The observed inter-individual variability in seroconversion timing (40% responders at week 6, 100% by week 16) suggests inconsistent intestinal uptake or processing efficiency across the population. This variability may reflect differences in gut microbiota composition, intestinal transit rates, or M-cell density among individuals—factors notoriously difficult to standardize in outbred livestock populations (29, 35). Strategies to reduce this variability could include enteric coatings to prevent premature gastric exposure, incorporation of additional adjuvants such as monophosphorylate lipid A (MPLA) or CpG oligodeoxynucleotides (36) to lower the threshold for immune activation, or optimization of fasting protocols to ensure consistent gastric emptying prior to dosing.

The 16-week observation window, while sufficient to demonstrate efficacy persistence through a standard rodent pubertal transition, does not address the longevity requirements of commercial livestock production, where immunocastration must persist for 5–6 months in pigs or potentially years in cattle. Long-term studies must evaluate the duration of antibody persistence, the potential for epitope spreading against carrier proteins, and the kinetics of immune exhaustion or tolerance development following chronic antigen exposure. Species translation presents specific challenges. Rodents possess gastric physiology and transit times distinct from monogastric livestock; the 12-hour intestinal transit in rats contrasts with approximately 48-hour retention in swine, potentially altering antigen exposure dynamics and release kinetics. Additionally, the enzymatic environment and microbiome of porcine or bovine intestines may degrade chitosan more aggressively than observed in murine models. Pilot studies in the target species must establish dose equivalency, as the 200 µg dose used here—effective in 300 g rats—would require substantial extrapolation for 100 kg pigs, necessitating formulation optimization to achieve dose-sparing (target ≤50 µg) while maintaining efficacy.

For additional requirements for specific article types and further information please refer to “Article types” on every Frontiers journal page.

## 5 Conclusion

This study provides robust proof-of-concept that alginate-chitosan microparticles encapsulating a recombinant GnRH-targeting fusion antigen (GF45) constitute an effective oral vaccine platform for immunocastration in male rodents. The formulation achieved endocrine suppression and reproductive tissue regression statistically equivalent to parenteral vaccination with Freund’s adjuvant, despite the formidable barriers of the gastrointestinal tract. The pH-responsive characteristics of alginate ensured gastric protection with subsequent intestinal targeting, while the chitosan-mediated mucoadhesive and immunostimulatory properties enabled evasion of oral tolerance and induction of sustained systemic IgG responses reaching 88.8% of injectable titers. Functional castration was complete, characterized by 95% reduction in circulating testosterone, 52% decrease in testicular weight, 75% reduction in testicular volume, and severe seminiferous tubule atrophy with Johnsen scores indicative of spermatogenic arrest.

Beyond immunological efficacy, this oral delivery strategy eliminates the handling stress, aversion behaviors, and acute cortisol responses associated with physical restraint and needle injection. This represents a significant welfare advancement for production animals and enables practical implementation in extensive grazing systems where individual handling is logistically challenging or economically prohibitive. The compatibility of microparticle formulations with voluntary administration via feed or drinking water opens realistic pathways for mass immunization in livestock operations and remote delivery for wildlife population management, overcoming the scalability limitations inherent to injectable vaccines.

Extending beyond immunocastration, this versatile platform accommodates diverse antigenic payloads through its modular polysaccharide matrix and adaptable GF45 fusion architecture. The system functions as a universal oral carrier for vaccines targeting enteric and respiratory pathogens, while enabling multivalent formulations that integrate fertility control with infectious disease prevention. Composed entirely of GRAS-status polymers (alginate and chitosan) with established safety profiles and manufactured via scalable aqueous processes compatible with standard pharmaceutical equipment, this technology circumvents the regulatory hurdles and toxicity concerns typically associated with synthetic nanocarriers or viral vectors, facilitating streamlined commercial deployment.

Collectively, these findings establish the foundation for a new generation of non-invasive oral veterinary vaccines aligned with contemporary demands for animal welfare, production sustainability, and reduced antimicrobial reliance. The demonstration that oral immunization can achieve functional efficacy equivalent to parenteral injection represents a paradigm shift for protein antigen delivery in veterinary preventive medicine, with broad applicability across monogastric species.

## Data Availability

The raw data supporting the conclusions of this article will be made available by the authors, without undue reservation.
